# Genome-wide analysis of *Rf-PPR-like* genes in *Nicotiana tabacum* and their potential roles in anther development

**DOI:** 10.3389/fpls.2025.1591130

**Published:** 2025-09-11

**Authors:** Mengting Wu, Yan Ji, Chengbei Zhang, Shuaibin Du, Ruqi Gong, Jun Wang, Jiayi Li, Qiu Zhong, Yuan Li, Aiguo Yang, Yazhi Cheng, Xingwei Zhang, Guoxiang Liu

**Affiliations:** ^1^ Tobacco Research Institute, Chinese Academy of Agricultural Sciences, Qingdao, China; ^2^ Leaf Tobacco Technology Extension Department, Deyang Company of Sichuan Provincial Tobacco Corporation, Deyang, China; ^3^ Institute of Tobacco Science, Fujian Provincial Tobacco Company of China National Tobacco Corporation, Fuzhou, China

**Keywords:** pentatricopeptide repeat (PPR) gene family, restoration of fertility like (RFL) gene, cytoplasmic male sterility (CMS), anther development, tobacco

## Abstract

Pentatricopeptide repeat (*PPR*) gene family is one of the largest gene families in higher plants. The Restoration of fertility like (*RFL*) clade of the family plays a crucial role in restoring fertility of cytoplasmic male sterility (CMS) lines in plants. Common tobacco (*Nicotiana tabacum* L.) is an important economic crop of which the CMS hybrids have been widely used in commercial cultivation. However, the restorer line of tobacco and the regulatory mechanism of fertility restoration remain elusive. In addition, *PPR* and *RFL* genes have not been illustrated in common tobacco. In this study, a total of 1002 *NtPPR* genes were identified, of which 27 *NtRFLs* belonging to P subfamily were demonstrated. The collinearity analysis showed that a total of 15 pairs of *NtRFL* genes had collinear relationship and unevenly distributed in 9 linkage groups. Cis-element analysis revealed that a large number of environmental stress and phytohormone response elements were located in the promoter of *NtRFLs*. By combining the RNA-seq and qPCR analysis, *NtRFL3* was further selected as the candidate gene due to its significantly higher expression at early anther development in the fertile line MF1. NtRFL3 was predicted to be localized in mitochondria and shared high sequence similarity with the known fertility-restorer PPR592 in petunia. Our results provided new gene targets for molecular breeding of tobacco restorer lines and for illustration of molecular mechanism on fertility restoration of plant CMS lines.

## Introduction

Pentatricopeptide repeat (PPR) proteins represent an extensive plant-specific protein family predominantly found in terrestrial plants (Small and Peeters, 2000). These sequence-specific RNA-binding proteins localize to semi-autonomous organelles and are crucial for plant growth and developmental processes (Liu et al., 2021). Following their initial discovery in Arabidopsis, subsequent studies have expanded our understanding of PPR proteins across various plant species. Structurally, PPR proteins comprise three distinct regions: an N-terminal targeting signal, a central tandem repeat domain containing 2-30 conserved motifs (each spanning 30-40 amino acids), and a C-terminal functional domain (Lurin et al., 2004). PPR gene family can be divided into P and PLS subfamily based on their motif structure (Han et al., 2020). The P subfamily contains P motifs common to all eukaryotes (Cheng et al., 2016), and PLS subfamily can be divided into four subgroups: PLS, E, E+, and DYW based on the different non-PPR domains at the C-terminus. The variable N-terminal region determines subcellular localization to either mitochondria or chloroplasts (Saha et al., 2007). Contemporary research has elucidated PPR proteins multifaceted roles in organellar gene expression regulation. These proteins mediate post-transcriptional processes including RNA editing, splicing, stabilization, and translation, while also influencing embryogenesis and chloroplast biogenesis (Hao et al., 2019; Hayes et al., 2015; Ichinose et al., 2012; Tavares-Carreón et al., 2008).

Cytoplasmic male sterility (CMS) is a maternal genetic trait in which mitochondrial gene abnormalities lead to stamen degeneration, pollen abortion or functional infertility in plants while pistils function normally (Baranwal et al., 2012). Some studies have found that CMS lines usually accumulate reactive oxygen species (ROS) in anthers and other tissues to form oxidative stress (Jiang et al., 2007), which is a direct cause of pollen abortion. Male sterility can be restored by the expression of restorer-of-fertility (*Rf*) genes, so that plants produce normal pollen (Dahan and Mireau, 2013). Numerous genes have been identified as *Rf* genes to play pivotal roles in mediating fertility restoration mechanisms in plants, such as the *Zea mays RF2* (Cui et al., 1996) and *Beta vulgaris RF1* (Kitazaki et al., 2015) belonging to the Aldehyde dehydrogenase and Peptidase-like family, respectively. The *Restoration of fertility like (RFL)* genes are also characterized as *Rfs*, derived from P subfamily of PPRs. Some *RFLs* can specifically regulate the transcription of male sterility genes in mitochondria and restore plant fertility (Fujii et al., 2011). Plant genomes generally encode 10-30 RFL proteins (Dahan and Mireau, 2013), most of which belong to P subfamily of PPRs. *Arabidopsis thaliana* has 26 *AtRFLs*, While there are 53 *BnRFL* and 38 *RFLs* in *Brassica napus* (Ning et al., 2020) and *Solanum tuberosum* (Ding, 2014), respectively. However, several RFL proteins, including Rf1 (Klein et al., 2005) in *Sorghum bicolor* and Rfm1 (Ui et al., 2015) in *Hordeum vulgare* have been functionally characterized as members of the PLS subfamily.

Current studies have identified the primary mechanisms by which Rfs restore plant fertility: Firstly, it involves expression suppression of sterility genes by Rfs binding and processing their RNA. The resulted metabolic reprogramming could compensate the cellular energy deficits caused by the expression of CMS-associated transcripts. In the radish Ogura CMS system (one CMS type in cruciferous crops), the Rf gene *orf687* encodes a protein that directly binds to the transcript of the sterility gene *orf138*, suppressing its expression at the post-transcriptional level (Desloire et al., 2003; Yamagishi et al., 2021). For rice CMS line (BT-type) caused by the mitochondrial gene *orf79*, *RF1a* mediates endonucleolytic cleavage of *atp6-orf79* mRNA, while *RF1b* promotes their rapid degradation, collectively restoring fertility of rice CMS lines (Akagi et al., 2004; Wang et al., 2006; Kazama et al., 2008).

Heterosis has been extensively exploited in *Nicotiana tabacum*, where F_1_ hybrids exhibit significantly enhanced growth vigor and stress resistance compared to the mid-parental values (Hancock and Lewis, 2017). The reliable production of these hybrids depends on CMS systems, which ensure seed purity by preventing paternal pollen contamination. The sterility genes originate from diverse sources, including wild species cytoplasm, natural mutation of sterile plants, and impaired coordination between nuclear and cytoplasm. For example, the sterility of tobacco CMS lines (designated as sua-CMS) is due to the wild species *Nicotiana suaveolens*-derived cytoplasm. Previous studies have identified mitochondrial respiratory chain-related genes as sterility genes which presented aberrant open reading frames (ORFs) leading to programmed cell death (PCD)-related pollen abortion and insufficient ATP synthesis in tobacco (Liu, 2022). However, the nuclear factors coordinating mitochondrial-nuclear interactions for fertility restoration remain poorly characterized.

With the deepening of the whole genome sequencing of tobacco, it provides a good basis for tobacco bioinformatic analysis. Ding (2014) systematically identified the PPR family members from *Nicotiana tomentosiformis* and cloned six *NtomRfs* (Ding, 2014). Through genome-wide analysis, we characterized PPR family members, especially *RFL* genes in cultivated tobacco, with their expression patterns validated by both transcriptomics and qPCR at different anther developmental stages. The findings would provide new gene targets for molecular breeding of tobacco restorer lines and theoretical foundation for illustration of regulatory network on fertility restoration of plant CMS lines.

## Materials and methods

### Identification of PPR family members in *Nicotiana tabacum* L.

The Hidden Markov Model (HMM) of PPR domain (PF01535) (Che et al., 2022) was downloaded from Pfam (http://pfam.xfam.org/). The HMM profile was used to identify all the potential PPR protein sequences through the Simple HMM Search in TBtools (Chen et al., 2020), using the genome data of *Nicotiana tabacum* cultivar ZY300 (Zan et al., 2025). The conserved domain of all identified NtPPRs were analyzed by NCBI Batch-CDD tools (http://www.ncbi.nlm.nih.gov/Structure/bwrpsb/bwrpsb.cgi) using the CDD-62466 position-specific scoring matrix (PSSMs) database. Key parameters were set as follows: E-value threshold set to 0.01, maximum matching sequences limited to 500, with remaining parameters maintained at default ones. The screened protein domains were further confirmed by SMART (http://smart.embl.de/smart/set_mode.cgi?GENOMIC=1) and Expasy (https://web.expasy.org/protparam) tools.

### Chromosomal localization and gene structure analysis

The chromosomal localization and gene structure information of *NtPPRs* was acquired in the annotation file of ZY300. The results were visualized by Map Gene 2 Chrom v2.1 (Chao et al., 2015) and Gene Structure Viewer in TBtools, respectively.

### Subcellular localization prediction

The Cell-Ploc (http://www.csbio.sjtu.edu.cn/bioinf/Cell-PLoc-2/), TargetP (http://www.cbs.dtu.dk/services/TargetP/), Predotar (https://urgi.versailles.inra.fr/predotar/predotar.html) were used to predict protein subcellular localization.

### Phylogenetic and conserved motif analysis

The PPR protein sequences of *Solanum lycopersicum* were downloaded from Sol Genomics Network (https://solgenomics.sgn.cornell.edu/). The sequences of five Rf-PPRs, RPF1 (*Arabidopsis thaliana*), Rf_PPR592 (*Petunia hybrida*), Rf1a (*Oryza sativa*), Rfo (*Raphanus sativus*), and PPR1 (*Capsicum annuum*), were downloaded from NCBI database (https://www.ncbi.nlm.nih.gov/). The protein sequences were aligned by MUSCLE using MEGA 11, and then subjected to genetic distance analysis using compute pairwise distance. Bootstrap analysis was selected with a default setting of 1000 replicates, and the p-distance method was chosen for the calculation. The phylogenetic analysis was performed by Neighbor-Joining (NJ) method with 500 bootstrap iterations. The ITOL (https://itol.embl.de/) was used to modify the output phylogenetic tree.

The MEME suite (http://meme-suite.org/tools/meme) was used to predict the conserved motifs of NtPPRs (Bailey et al., 2009). The seven conserved motifs of Arabidopsis were used as primary sequences to discover NtPPR motifs.

### Identification and physicochemical property analysis of NtRFLs

The protein sequences of AtRFL1-AtRFL26 and Rf-PPR592 were downloaded from NCBI (https://www.ncbi.nlm.nih.gov/), and were used for BLAST searches against the protein sequences of ZY300 in TBtools with E-value<e^-100^. The isoelectric point, molecular weight, amino acid number, instability index, aliphatic index and grand average of hydropathicity of NtRFLs were analyzed by ProtParam (https://web.expasy.org/computepi/).

### Collinearity analysis of NtRFLs

Genomic data of *Solanum tuberosum* and *Arabidopsis thaliana* were downloaded from Sol Genomics Network and TAIR (https://www.arabidopsis.org), respectively. The One Step MCScanX in TBtools was used to analyze gene duplication events. The syntenic relationship of *RFL* genes between tobacco and the other two species was determined using the Dual Synteny Plotter tool in TBtools. The results were visualized by the Advanced circos in TBtools.

### Prediction of cis-acting elements in *NtRFLs*

The promoter sequence of *NtRFLs* (2000 bp upstream sequences of CDS) were extracted by TBtools according to the annotation file of ZY300. The PlantCARE online tool (http://bioinformatics.psb.ugent.be/webtools/plantcare/html) was used to predict the cis-acting elements of promoter, and Rstudio was used for statistical and visual analysis of each element.

### Cytological observation of flower buds in tobacco cultivars with different fertility

The 2-3 mm, 3-5 mm, 5-7 mm of flower buds, corresponding to sporogenous cell-microsporocyte period, meiosis-tetrad period, and the mid uninucleate period, were collected and subsequently embedded into paraffin according to Yuan (Yuan et al., 2018). The toluidine blue stained sections was observed using the inverted microscope.

### Total RNA extraction, cDNA synthesis and qPCR analysis

Flower buds (2-3 mm, 3-5 mm, 5-7 mm in size) were sampled from three tobacco varieties: the fertility line F609 and two sterile line MS609 (which possesses stamens but lacks pollen grains), and MSG28 (stamenless). Three biological replicates were performed for each line. Samples were stored at -80°C after quick freezing in liquid nitrogen.

Total RNA with three biological replicates were extracted using TRNpure reagent (Nobelab, China) according to the manufacturer’s instructions. 1 μg RNA was used for cDNA synthesis using Evo M-MLV Mix Kit with gDNA Clean for qPCR (AccurateBiology, AG11728, China). All reactions were performed with two technical replicates using SYBR Green Premix Pro Taq HS qPCR Kit (AccurateBiology, AG11701, China), with primers indicated in **Supplementary Table S1**. qPCR was conducted on the LightCycler 96 Instrument. Relative gene expression was normalized to the expression level of *Actin*.

### Transcriptome analysis

RNA samples extracted from 2-3 mm flower buds were sequenced by the High-Throughput Sequencing Instruments using the Illumina NovaSeq 6000 (illumina, USA) platform, obtained an average yield of 5.9 Gb data per sample. Sequencing adapter contamination, reads with N bases, and low-quality reads were removed using fastp (version 0.19.7). Clean reads were aligned against ZY300 reference genome using the Hisat2 (V2.0.5) software (Mortazavi et al., 2008). Gene expression levels were log-transformed and normalized using DESeq2 (1.20.0) software. The FPKM value of *NtRFL* genes were used to characterize their expression patterns.

## Results

### Identification of PPR family members in *Nicotiana tabacum* L.

**Figure 1:**
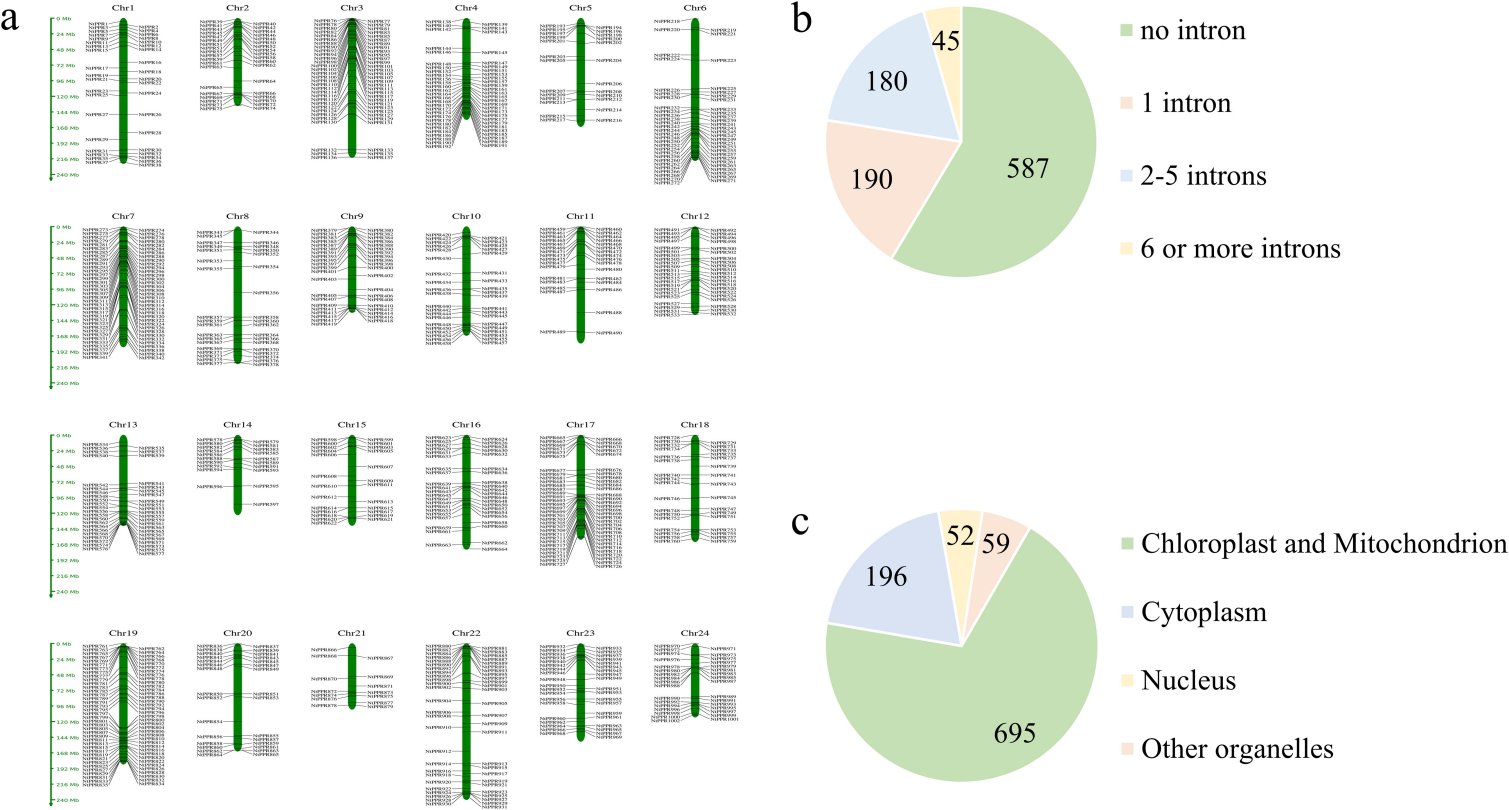
Chromosomal distribution, gene structure and subcellular localization of PPR genes in tobacco. **(a)** Chromosomal localization of *NtPPR* genes. **(b)** Number of *NtPPR* genes with different intron numbers. **(c)** Number of NtPPRs with predicted subcellular localization.

Through a comprehensive analysis combining hidden Markov model (HMM) profiling and conserved domain verification, we systematically identified 1002 pentatricopeptide repeat (PPR) genes in the tobacco genome. These *NtPPR* genes were chromosomally mapped and numerically designated from *NtPPR1* to *NtPPR1002* according to their genomic positions (**Figure 1a**). Structural characterization revealed distinct intron-exon patterns: 58.5% of *NtPPR* genes existed as single-exon structures, 19.0% contained one intron, while the remaining 22.5% possessed two or more introns (maximum 18 introns per gene) (**Figure 1b**). Prediction of subcellular localization showed that 69.36% of NtPPRs were localized either in chloroplasts or in mitochondria (**Figure 1c**).

### Phylogenetic analysis and classification of NtPPRs

**Figure 2:**
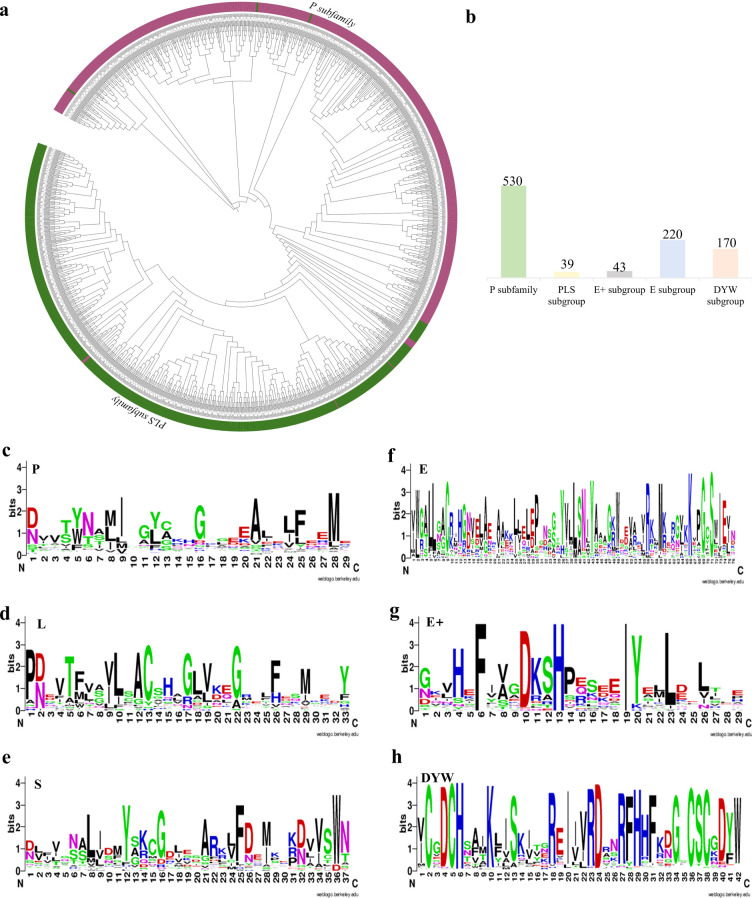
Phylogenetic relationships and conserved motif analysis among the NtPPR family genes. **(a)** PPR phylogenetic tree. 1002 NtPPRs and 471 SlPPRs were aligned. P and PLS subfamilies were highlighted in purple and green, respectively. **(b)** Number of NtPPR proteins belonging to the P subfamily and PLS subgroups. **(c-h)** Conserved motif analysis among the NtPPR family genes. The height of characters correlates to the conservation of the amino acid, the higher the character height, the more conservative of the amino acid.

To clarify the subfamilies of NtPPRs, phylogenetic tree was constructed using the identified 1002 NtPPRs and 471 known *Solanum lycopersicum* PPR proteins. The analysis revealed clear separation into two major subfamilies: the P subfamily (530 members) and PLS subfamily (472 members) (**Figure 2a**). Through protein conservation analysis, we identified six conserved motifs that enabled further subdivision of the PLS subfamily (**Figure 2c‐h**). The PLS subfamily was subsequently divided into four distinct subgroups: PLS (39 members), E (220), E+ (43), and DYW (170), as shown in (**Figure 2b**).

### Identification and physicochemical property analysis of tobacco RFL-PPR members

We performed the *NtRFLs* identification based on their homology with Arabidopsis RFLs and petunia *Rf-PPR592*. Among the 26 *AtRFLs*, 9 had no homologs in tobacco, including *AtRFL1*, *AtRFL8*, *AtRFL10*, *AtRFL19*, *AtRFL20*, *AtRFL22*, *AtRFL23*, *AtRFL24*, and *AtRFL26*. By using *Rf-PPR592* as a reference, 20 *NtRFLs* were identified. Combining both blast results, a total of 27 *NtRFLs* were identified in tobacco all belonging to P subfamily. These genes were designated as *NtRFL1* to *NtRFL27*. The molecular weight of NtRFLs ranged from 442 to 148.8 KDa, with NtRFL16 and NtRFL22 as smallest and largest proteins, respectively. The theoretical isoelectric points of NtRFLs varied from 5.94 to 9.03, with 6 NtRFLs as acidic proteins having pI values below 7, while the rest were alkaline. The instability index of NtRFL proteins were all below 40, suggesting their high stability. The coefficients of lipid solubility differed from 92.09 to 104.31, and the negative GRAVY values of 10 RFL proteins demonstrated their hydrophilic nature (**Table 1**).

**Table 1:** Hysicochemical property analysis of NtRFLs.

Gene name	Number of amino acid/aa	Molecular weight/Da	Isoelectric point	Instability index	Aliphatic index	Grand Average of hydropathicity
NtRFL1	717	80568.75	8.06	27.13	98.03	0.012
*NtRFL2*	533	60450.69	6.26	27.73	97.99	0.047
*NtRFL3*	606	68360.64	6.58	29.95	98.61	0.046
*NtRFL4*	537	60977.67	7.79	26.62	99.40	0.046
*NtRFL5*	726	81698.59	7.87	27.08	92.09	-0.088
*NtRFL6*	726	81401.30	7.06	26.48	94.12	-0.057
*NtRFL7*	591	66916.30	8.71	26.16	93.96	0.008
*NtRFL8*	595	67672.27	8.60	30.90	96.47	0.005
*NtRFL9*	678	77216.99	9.03	29.82	97.57	0.046
*NtRFL10*	595	68066.58	8.43	34.36	97.58	-0.042
*NtRFL11*	595	67581.02	8.44	32.12	98.10	0.013
*NtRFL12*	578	65709.67	6.98	28.11	96.61	0.014
*NtRFL13*	625	71347.20	8.18	32.99	95.44	-0.039
*NtRFL14*	590	66727.86	8.50	29.07	96.49	0.047
*NtRFL15*	414	47070.87	8.81	38.90	96.93	0.019
*NtRFL16*	389	44229.58	6.33	30.45	97.20	-0.089
*NtRFL17*	469	53558.56	5.94	27.58	104.31	-0.012
*NtRFL18*	599	67112.96	8.32	27.94	92.37	-0.055
*NtRFL19*	532	60816.20	8.27	33.95	99.47	-0.013
*NtRFL20*	701	80608.06	8.33	37.28	95.93	-0.057
*NtRFL21*	577	65507.92	8.69	30.99	98.11	0.04
*NtRFL22*	1312	148766.90	7.60	32.01	100.82	0.049
*NtRFL23*	580	65840.99	8.44	27.71	101.14	0.017
*NtRFL24*	542	61684.29	8.05	39.87	98.73	0.076
*NtRFL25*	605	67838.07	8.28	28.75	92.26	-0.023
*NtRFL26*	727	81838.44	8.26	29.57	97.50	0.036
*NtRFL27*	596	67486.51	6.06	32.31	99.93	0.049

### The collinearity analysis of tobacco RFLs

**Figure 3:**
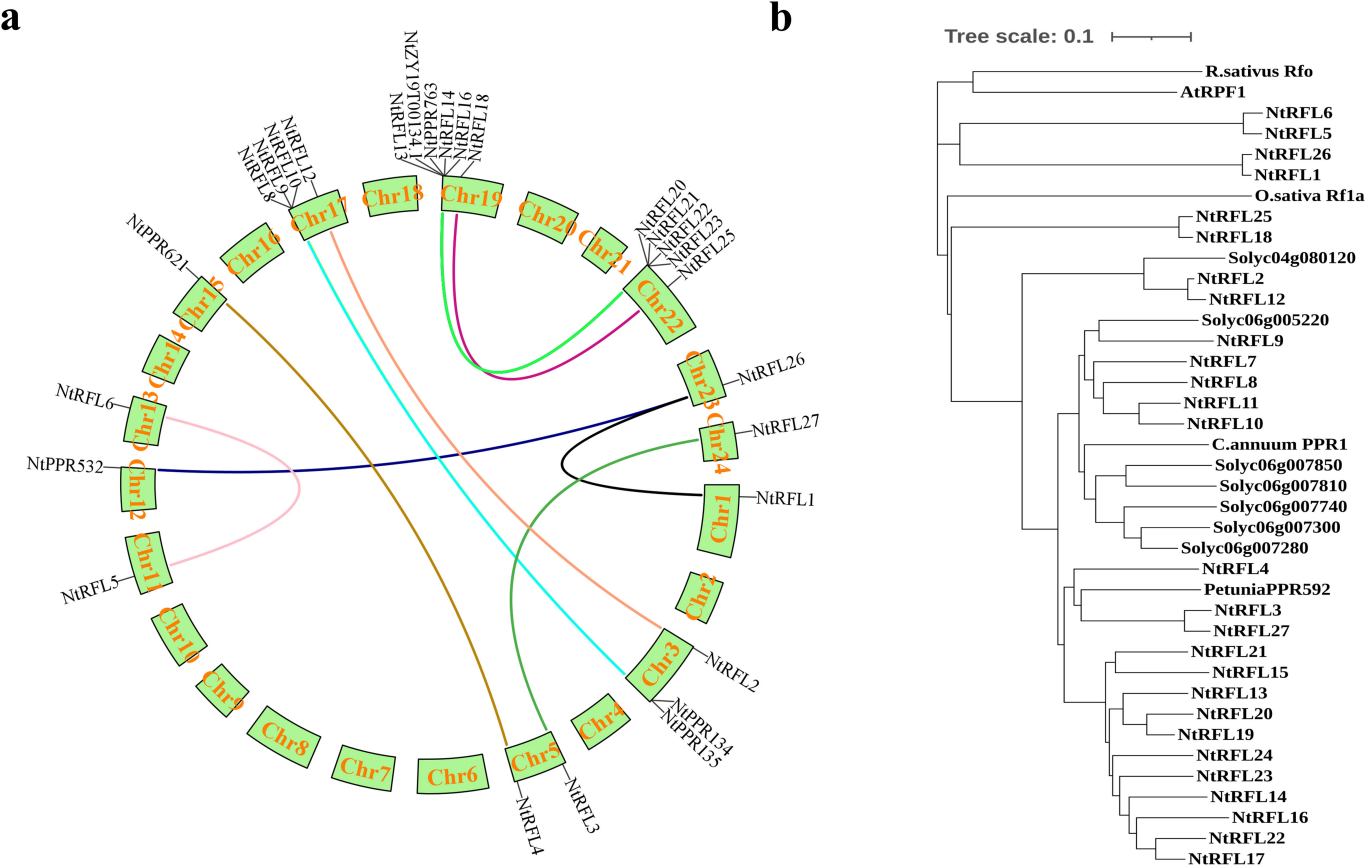
Evolutionary relationship of RFL genes in tobacco. **(a)** Distribution of collinear RFL gene pairs on tobacco chromosomes. The different colored lines indicate collinear *RFL* gene pairs. **(b)** Phylogenetic tree of Rf proteins in multiple species. Rs, *Raphanus sativus*; Os, *Oryza sativa*; At, *Arabidopsis thaliana*; Ca, *Capsicum annuum*; Ph, *Petunia hybrida*; Solyc, *Solanum lycopersicum*.

To investigate the evolutionary patterns of tobacco *RFLs*, we performed collinearity analysis of gene duplication events. The results (**Figure 3a**) revealed 15 collinear gene pairs unevenly distributed across 9 linkage groups. There were 6 *NtRFLs* distributed on Chr19, 4 *NtRFLs* on Chr17, and only 1 *NtRFL* on Chr1, Chr11, Chr12, Chr15 and Chr24 respectively. *NtRFL26* had collinearity with both *NtRFL1* and *NtPPR532* indicating that they may evolve distinct roles due to gene replication events. For the interspecific collinearity of *RFLs*, it showed that no collinearity was found in *RFLs* between tobacco and Arabidopsis. While *NtRFL9* forms homologous gene pairs with both *Solyc06g007740* in tomato and *Soltu06g002440* in potato suggesting potential conserved functions of these genes between different species (**Table 2**).

**Table 2:** Collinear gene pairs in the tobacco, tomato and potato.

Pairs	Chromosome	Gene	Chromosome	Gene
1	Chr1	*NtRFL1*	Chr23	*NtRFL26*
2	Chr5	*NtRFL3*	Chr24	*NtRFL27*
3	Chr5	*NtRFL4*	Chr15	*NtPPR621*
4	Chr11	*NtRFL5*	Chr13	*NtRFL6*
5	Chr17	*NtRFL8*	Chr3	*NtPPR134*
6	Chr17	*NtRFL9*	Chr3	*NtPPR135*
7	Chr17	*NtRFL10*	Chr3	*NtPPR134*
8	Chr17	*NtRFL12*	Chr3	*NtRFL2*
9	Chr19	*NtRFL13*	Chr22	*NtRFL20*
10	Chr19	*NtRFL14*	Chr22	*NtRFL21*
11	Chr19	*NtRFL16*	Chr22	*NtRFL22*
12	Chr19	*NtRFL18*	Chr22	*NtRFL25*
13	Chr22	*NtRFL20*	Chr19	*NtZY19T00134*
14	Chr22	*NtRFL23*	Chr19	*NtPPR763*
15	Chr23	*NtRFL26*	Chr12	*NtPPR532*
16	Chr17	*NtRFL9*	Chr6	*Solyc06g007740*
17	Chr17	*NtRFL9*	Chr6	*Soltu06g002440*

We also examined the homology of *NtRFLs* with various known *Rfs* in multiple species (**Figure 3b**). The results demonstrated that the *RFL* genes of tobacco predominantly clustered with those of tomato, pepper, and petunia, all belong to *Solanacea*, while being distant from those of rice and *Arabidopsis*. Among them, 14 *NtRFLs* were clustered with petunia *PPR592* which is encoded by a tandem array of 14 *PPR* motifs and is able to restore fertility to CMS plants by decreasing the accumulation of petunia mitochondrial fused gene (PCF) (Bentolila et al., 2002). This indicates that they may be the orthologs of *PPR592* and play similar roles in regulating plant fertility.

### Analysis of cis-acting elements of *NtRFLs*

**Figure 4:**
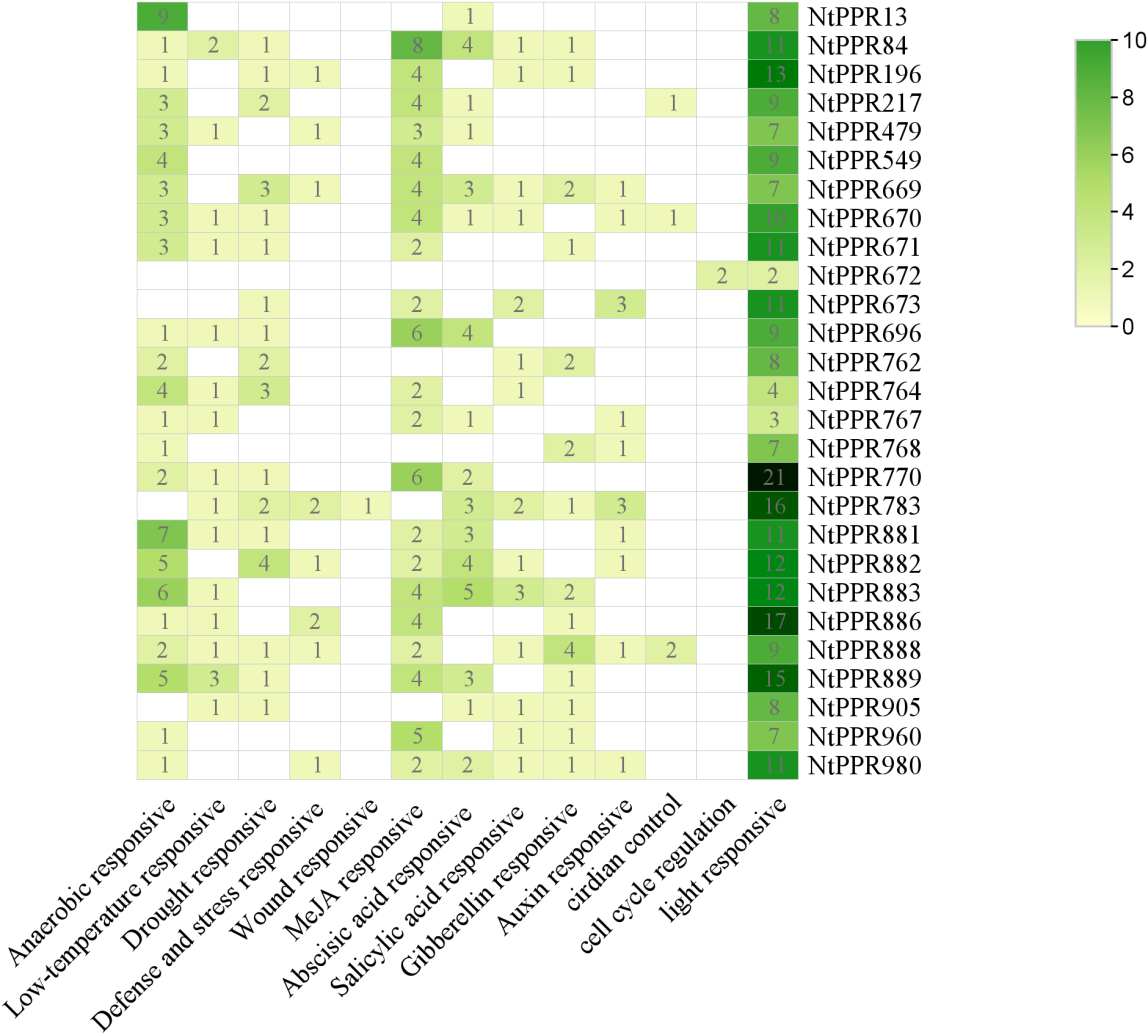
Prediction results of cis-acting elements of NtRFL genes. The number represents the element numbers in each category contained in *NtRFLs*.

To further explore the regulatory mechanism of *RFLs* gene expression in tobacco, we first performed cis-acting element analysis of their promoters (**Figure 4**). After removing non-functional elements, 13 types of cis-acting elements were identified in this study. These elements were mainly related to environmental stress and phytohormone responses, which perhaps collectively regulate the expression of the *NtRFLs* in tobacco.

Several thermo-sensitive male-sterile genes have been cloned and reported. However, how the environmental signals contribute to the fertility restoration remains unclear. We found that tobacco *RFL* genes, especially *NtRFL2* and *NtRFL24*, contained both low-temperature and light responsive elements, suggesting their potential role in pollen fertility recovery in a thermo-mediated way. Additionally, there are many cis-elements in response to phytohormones such as jasmonic acid, abscisic acid, salicylic acid, gibberellin and auxin. Zang et al. (2023) demonstrated that excessive activation of auxin signaling may inhibit pollen development, while inhibiting auxin signaling partially promoted pollen development in CMS-D2 cotton (Zang et al., 2023). Moreover, the fertility of Rice could be restored by applying exogenous methyl jasmonate (Pak et al., 2021). Therefore, the genes possessing hormone-responsive cis-elements are potential targets for studying the regulation of pollen fertility in response to various phytohormones in tobacco.

### Cytological observation of flower buds in tobaccos with different fertilily

**Figure 5:**
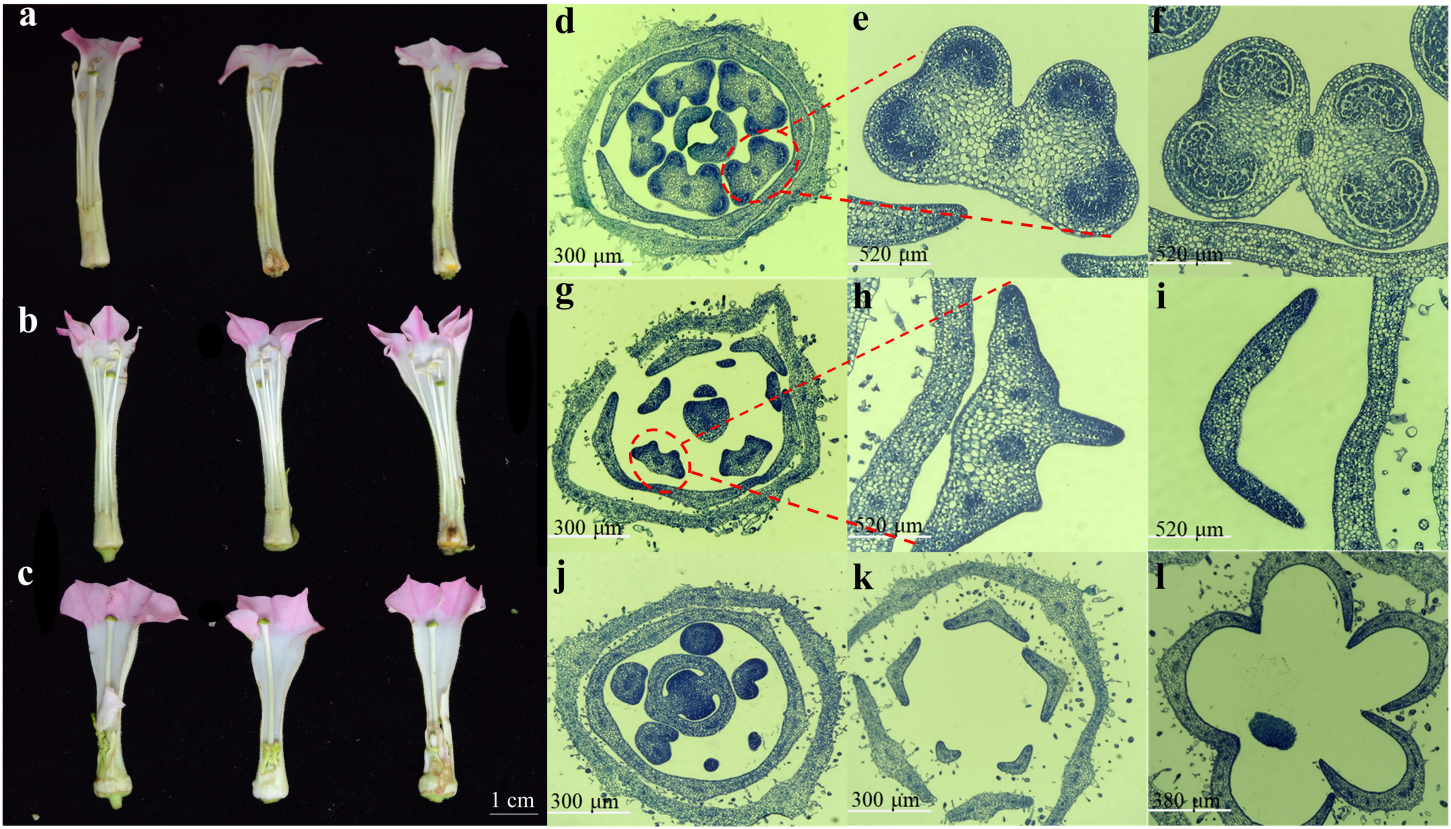
Morphological and cytological observation of anther development in three different tobacco lines. Floral phenotypes of **(a)** MF1 (fertile line), **(b)** CMS1 (semi-sterile line which possesses stamens but lacks pollen grains), and **(c)** CMS2 (the sterile line which is stamenless). **(d-l)** The cross sections of flower buds at the sporogenous cell-microsporocyte period, meiosis-tetrad period, and the mid uninucleate stage in MF1 (d-f), CMS1 **(g-i)** and CMS2 **(j-l)**, respectively.

To explore the expression profile of *NtRFLs* during anther development, three tobacco lines displaying diverse fertility as well as collected at different stages were used. The anther of fertile line MF1 developed normally with distinct cytological structures in each stage. Firstly, a transition stage including both sporogenous cell and microsporocyte periods was presented in the small buds (2-3 mm) of MF1. This was followed by meiosis and tetrad periods when the tapetum was degradated gradually in the buds of 3-5 mm. At the mid uninucleate stage exhibited in flower buds of 5-7 mm, the tapetum was almost disappeared, and the anther became mature with large number of pollen grains inside (**Figures 5a, d–f**). In contrast with MF1, the semi-sterile line CMS1 showed an irregular structure of tapetum resulting into disappeared pollen sacs (**Figures 5g–h**). At the mid unicucleate stage, a lamellar structure without pollen grains was formed as it’s shown in **Figures 5b, i**. Instead of showing three distinct stages described above, sterile line CMS2 with small buds was still at an earlier time point when the stamen primordium haven’t differentiated into sporogenous cells (**Figure 5j**). The cells were degenerated later and no stamens formed completely (**Figures 5c, k–l**).

### Expression analysis of *NtRFLs* in tobaccos with different fertilily

**Figure 6:**
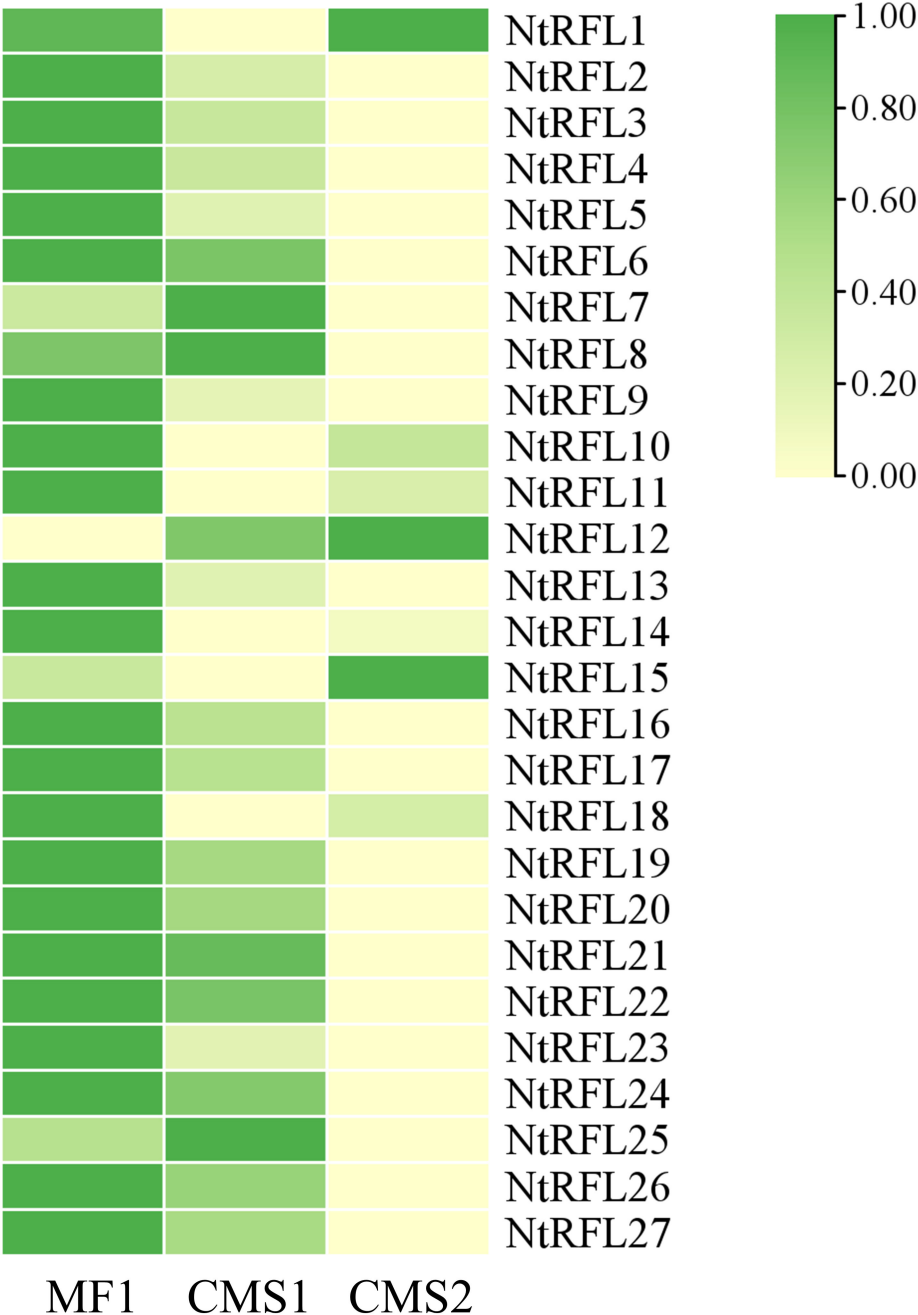
RNA-seq analysis of NtRFL genes in tobacco lines with different fertility. The up- and down-regulation were presented as green and yellow, respectively. MF1, the fertile line; CMS1, the semi-sterile line which possesses stamens but lacks pollen grains; CMS2, the sterile line which is stamenless.

Previous studies have found that cotton restoration gene *n-PPR-1*, *n-PPR-2* is specifically expressed in early anther development (Gao et al., 2022). It is speculated that fertility related genes are expressed at sporogenous cell-microsporocyte period or even earlier for determining the fate of embryonic cells. We therefore analyzed the transcriptome data in flower buds of 2-3 mm from the above lines. It was shown that 21 out of 27 *NtRFLs* were highly expressed in fertile line MF1, while expressed in lower level in CMS sterile lines (**Figure 6**).

**Figure 7:**
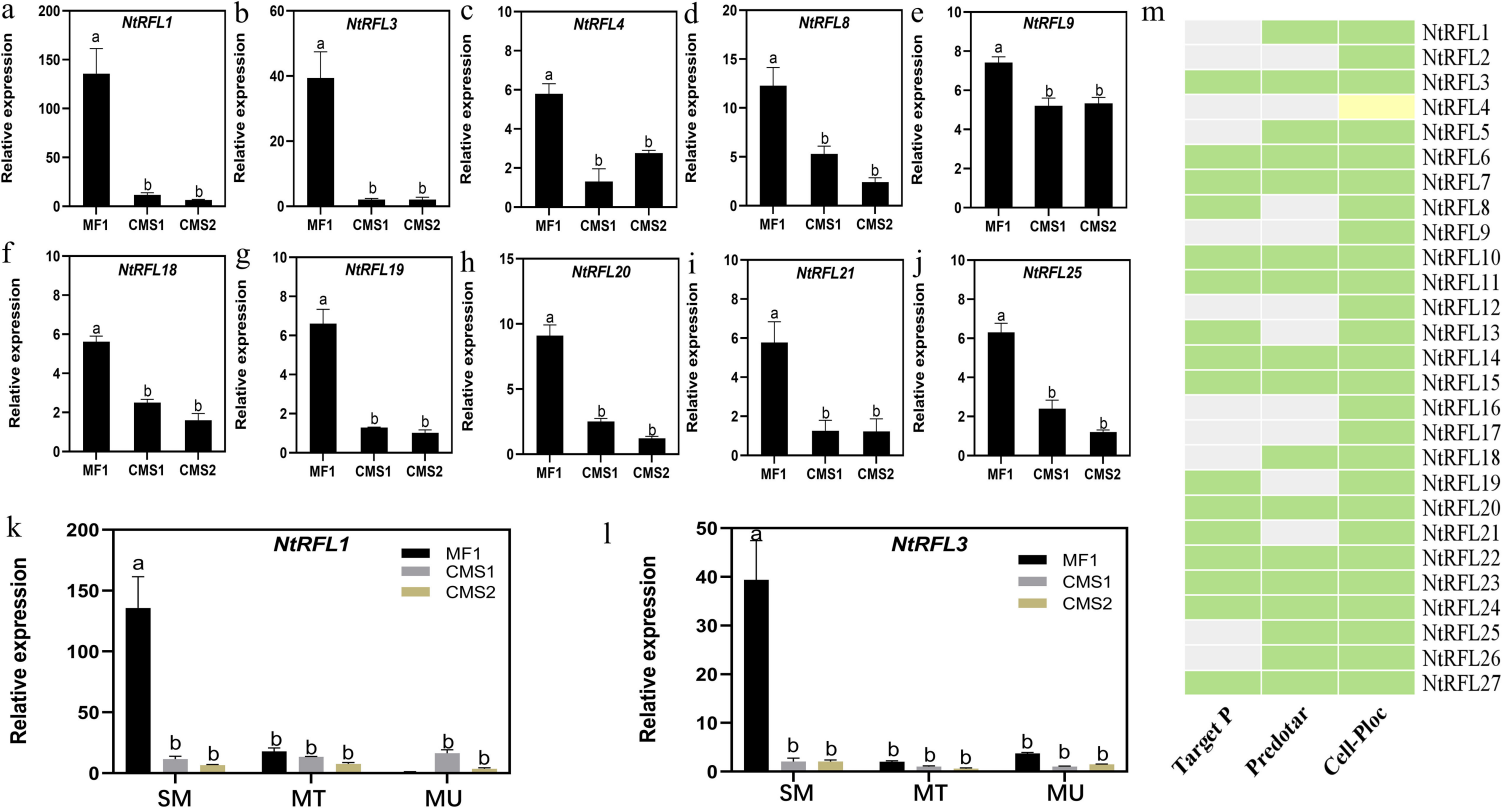
qPCR analysis and subcellular localization prediction of NtRFLs. **(a-l)** The expression of *NtRFLs* at sporogenous cell-microsporocyte stage **(a-j)** or at all three developmental stages **(k-l)** in MF1 (fertile line), CMS1 (semi-sterile line which possesses stamens but lacks pollen grain) and CMS2 (the sterile line which is stamenless). SM, sporogenous cell-microsporocyte stage; MT, meiosis-tetrad stage; MU, mid uninucleate stage. **(m)** Subcellular localization of NtRFLs. The green denoted mitochondria; the yellow are chloroplasts; the grey meant unsure.

To validate the RNA-seq results, we carried on qPCR analysis of all *NtRFLs* and found that 10 genes had higher expression in MF1 compared to CMS lines (**Figures 7a–j**). Whether their expression were specific to early time point was tested in samples including all three stages mentioned above. The results demonstrated that *NtRFL1* and *NtRFL3* were highly active at sporogenous cell-microsporocyte period of MF1, while there was no or less expression shown in CMS lines (**Figures 7k–l**).

RFLs can specifically regulate the transcription of male sterility genes in mitochondria and restore plant fertility (Fujii et al., 2011). The subcellular localization prediction of TargetP and Predotar software were proved to be highly consistent with that of fluorescence protein localization experiments (Lurin et al., 2004). Here, three different web tools were used for NtRFL1 and NtRFL3 subcellular localization prediction. It was demonstrated that they may localize in mitochondria (**Figure 7m**). As NtRFL3 had consistent expression patterns in both transcriptome data and qPCR analysis, and shared high similarity (79%) with petunia PPR592 which has been functionally characterized as a fertility restorer gene (**Figure 3b**), it was further selected as the candidate gene and speculated as a key regulator in early anther development and determination of plant fertility.

## Discussion

Taking the advantage of next-generation sequencing technology, it is possible to identify and study gene families at the whole genome-wide level. At present, 441 PPR genes have been identified in Arabidopsis (Lurin et al., 2004), 491 PPRs in rice (O'Toole et al., 2008), 626 PPRs in poplar (Xing et al., 2018), 1079 PPRs in *Brassica napus* (Ning et al., 2020), 181 PPRs in grape (Che et al., 2022) and 105 in moss (Sugita et al., 2013). Common tobacco is derived from the natural genome doubling after hybridization between *Nicotiana tomentosiformis* and *Nicotiana sylvestris* (Clarkson et al., 2005). In this study, we identify 1002 *PPR* genes in allotetraploid common tobacco which is more than twice the number of diploid tomato (471 PPR) and *Nicotiana tomentosiformis* (487 PPR) (Ding, 2014), both belonging to Solanacea. Chromosome localization analysis showed that PPR genes distributed more widely on chromosomes and did not appear in clusters, so it was speculated that the functions of tobacco PPR gene family members may be more complex and diverse.

The Rf-PPR proteins constitute evolutionarily unique protein subgroups in the PPR family of angiosperms (Dahan and Mireau, 2013). In this study, 27 *NtRFLs* were identified in *N.tabacum* cultivar ZY300 through bioinformatics tools, all of which belonged to the P subfamily. However, the number of *NtRFLs* was much less than 1/10 of the members of the P subfamily which was indicated in previous studies. This may be related to the fact that *Solanaceae* species including tobaccos experienced a whole genome triploid event (Gebhardt, 2016), resulting in the loss of some *RFL* genes.

*Rfs* is a high linkage on chromosomes (Lee et al., 2014). Studies presented that multiple genetically linked *Rf* genes restoring identical CMS localized within the same restorer locus. For instance, two identified *Rf* genes found to colocate in *Mimulus guttatus.* The genetically linked *Rf1* and *Rf2* mapped in *Mimulus guttatus* were found to reside in chromosomal loci containing 12 and 6 *Rf-like* genes, respectively (Barr and Fishman, 2010). Similarly, the sorghum *Rf5* locus was mapped to a 584-kb DNA fragment where researchers identified a cluster of 6 *PPR* exhibiting strong homology with the rice *Rf1a* (Jordan et al., 2011). Here, the synteny analysis revealed clustered distribution patterns of *NtRFLs* along chromosomes, with 15 *NtRF*L gene pairs unevenly distributed across 9 linkage groups.

Anther morphogenesis exhibits direct correlation with plant fertility. Developmental defects in anthers at any growth stage may lead to male sterility. CMS plants demonstrate diverse phenotypes in their impaired male reproductive organs, such as abnormal anther morphogenesis or defects in functional pollen development. In rice CMS systems, immature pollen grains show CMS-type-specific developmental arrest, with cytological differences in starch accumulation levels. PCD is crucial for normal anther formation, especially in tapetum and pollen sac wall cells (Ma, 2005). Premature or delayed tapetal PCD has been documented to induce male sterility through disrupted sporopollenin deposition and pollen wall patterning (Ji et al., 2013). In this study, we observed that tobacco CMS2 line remained undifferentiated with visible stamen primordium in 2-3 mm flower buds. The following developmental process ceased and the stamens were found to be completely degenerated when its flower became mature. Conversely, intermediate type CMS1 exhibited an undeveloped anther with lighter color and not filled with pollen grains, which was already noticed at the meiosis-tetrad period. These findings corroborate previous research indicating that stamens in male-sterile lines exhibit developmental anomalies at the floral bud stage, manifesting as tissue fusion with the pistil base and failure to undergo further differentiation (Cui et al., 2023).

Research evidence indicates that early anther developmental stages are critical for microspore fertility. Cytological studies have shown that pollen abortion predominantly initiates during the microspore mother cell differentiation phase and tetrad formation stage, highlighting the importance of early anther development in determining the fate of plant fertility (Li, 2015). The expression pattern analysis in this study showed that *NtRFL1* and *NtRFL3* were highly active at sporogenous cell-microsporocyte period of MF1, but near half of *NtRFLs* also had high expression in tobacco CMS lines. This has been seen in a previous study where the expression of *NtomRf* in MS K326 is higher than that in the maintainer line (Ding, 2014). It is hypothesized that the *Rf* gene remains expressed in male-sterile lines, but fails to be translated into functional proteins or form effective complexes.

Notably, gametophyte development involves exceptional energy demands. Tapetal cells exhibit mitochondrial densities up to 40-fold higher than somatic cells (Duroc et al., 2005). Such extraordinary mitochondrial proliferation likely provides essential energy support for pollen development. However, CMS associated gene products disrupt mitochondrial biogenesis, leading to metabolic dysfunction. This interference ultimately triggers premature PCD in the tapetum, resulting in pollen abortion. Studies demonstrates that all characterized restorer PPR proteins localize to mitochondria and function by specifically reducing the accumulation of CMS-associated mitochondrial RNA or protein. Furthermore, the protein-protein interaction network of Arabidopsis RFL proteins suggests their potential recruitment of chaperone partners (Hölzle et al., 2011; Fujii et al., 2016). RFL proteins lack intrinsic endonuclease activity, indicating their functional dependence on forming multimeric complexes with auxiliary protein factors. At present, the cloning and regulatory mechanism of CMS restoration genes in *Nicotiana tabacum* have not yet been realized. The molecular mechanism of how the *NtRFL* regulates the expression of CMS genes in mitochondria and affects plant fertility is still unknown, which requires further investigation.

## Data Availability

All relevant data is contained within the article: The original contributions presented in the study are included in the article/**Supplementary Material**, further inquiries can be directed to the corresponding author.
